# Exponential Clonal Expansion of 5-Fluorocytosine–Resistant *Candida tropicalis* and New Insights into Underlying Molecular Mechanisms

**DOI:** 10.3201/eid3105.241910

**Published:** 2025-05

**Authors:** Nissrine Abou-Chakra, Karen Marie Thyssen Astvad, Jan Martinussen, Amalie Sofie Eilsø Munksgaard, Maiken Cavling Arendrup

**Affiliations:** Author affiliations: Statens Serum Institut, Copenhagen, Denmark (N. Abou-Chakra, K.M.T. Astvad, A.S.E. Munksgaard, M.C. Arendrup); Technical University of Denmark, Kgs Lyngby, Denmark (J. Martinussen); University of Copenhagen, Copenhagen (A.S.E. Munksgaard, M.C. Arendrup); Rigshospitalet, Copenhagen (M.C. Arendrup)

**Keywords:** antimicrobial resistance, fungi, flucytosine resistance, Candida tropicalis, clonal spread, emerging resistance, Denmark

## Abstract

In 2022, we initiated systematic 5-fluorocytosine susceptibility testing of *Candida* spp. isolates in Denmark; we observed a bimodal MIC distribution in *C. tropicalis*, with MICs >16 mg/L in half the isolates. This study investigates the epidemiology and molecular mechanisms of 5-fluorocytosine resistance in *C. tropicalis*. We analyzed 104 *C. tropicalis* isolates from 3 time periods, alongside 353 *C. albicans* and 227 *C. glabrata* isolates from 2022. We determined MICs using EUCAST E.Def 7.3. Sequencing of *FCY2* (purine-cytosine permease), *FCY1* (cytosine deaminase), *FUR1* (uracil phosphoribosyl transferase), and *URA3* (orotidine-5′-phosphate decarboxylase) genes revealed FCY2 alterations—E49X (30/32), Q7X (1/32), and K6NfsX10 (1/32)—in resistant *C. tropicalis* strains. We found a URA3 alteration, K177E, in both susceptible and resistant strains. Microsatellite genotyping showed that all *C. tropicalis* isolates with E49X were clonally related. The marked increase in resistance, driven by the clonal spread of E49X, necessitates further research into virulence and environmental factors.

*Candida tropicalis* is a globally distributed opportunistic pathogen that can cause invasive infections in immunocompromised and predisposed patients (*1*,*2*). It ranks among the top 4 *Candida* species responsible for candidemia in Denmark, after the 2 most prevalent species, *C. albicans* and *C. glabrata* (*3*). However, *C. tropicalis* is more prevalent in southern Europe countries and Asia, where clonal spread in hospital settings has been reported (*4*–*6*).

5-fluorocytosine is an antifungal agent that targets nucleic acid and protein synthesis. As a prodrug, it undergoes several chemical modifications to become active (*7**–**9*) (Table 1). 5-fluorocytosine is primarily used for cryptococcal meningitis and difficult-to-treat central nervous system, eye, or bone infections caused by *Candida* species, and in combination with amphotericin B to avoid resistance selection (*7*); it is rarely included in routine susceptibility of *Candida* species.

**Table 1:** Metabolism and mode of action of 5-fluorocytosine in study of clonal expansion of 5-fluorocytosine–resistant *Candida tropicalis*, Denmark*

Step	Process	Enzyme/mechanism	Outcome
Uptake	5-fluorocytosine is taken up into fungal cells	Transport protein purine-cytosine permease (encoded by *FCY2;* primary uptake mechanism)	Initiates the antifungal activity of the metabolic pathway
Conversion to 5-FU	5-fluorocytosine is metabolized into 5-FU	Cytosine deaminase (encoded by *FCY1*); selective action due to lack of enzyme in mammalian cells	5-fluorocytosine → 5-FU
Formation of 5-FUMP	5-FU is converted into 5-FUMP	Uracil phosphoribosyl transferase (encoded by *FUR1*)	5-FU → 5-FUMP
Phosphorylation to 5-FUDP	5-FUMP is phosphorylated to 5-FUDP	Phosphorylation by specific kinases (e.g., uridine monophosphate kinase)	5-FUMP → 5-FUDP
Formation of 5-FUTP	5-FUDP is further phosphorylated to 5-FUTP	Phosphorylation reactions	5-FUDP → 5-FUTP; integration into RNA leads to dysfunctional RNA and inhibition of RNA synthesis
Formation of 5-FdUDP	5-FUDP is reduced to 5-FdUDP	Ribonucleotide reductase	5-FUDP → 5-FdUDP
Formation of 5-FdUTP	5-FdUDP is phosphorylated to 5-FdUTP	Phosphorylation reactions	5-FdUDP → 5-FdUTP; incorporation into DNA inhibits DNA synthesis and repair
Dephosphorylation to 5-FdUMP	5-FdUTP is dephosphorylated to 5-FdUDP, which is subsequently reduced to 5-FdUMP, inhibiting TS	Dephosphorylation reactions Ribonucleotide reductase	The irreversible inhibition of TS leads to depletion of dTMP and dTTP, disrupting nucleotide pools, leading to DNA damage, potential apoptosis, and "thymine-less death"

*5-FdUMP, 5-fluoro-deoxyuridine monophosphate; 5-FU, 5-fluoro-uracil; 5-FUMP, 5-fluoro-uridine monophosphate; 5-FUDP, 5-fluoro-uridine diphosphate; 5-FUTP, 5-fluoro-uridine triphosphate; 5-FdUDP, 5-fluoro-deoxyuridine diphosphate; 5-FdUTP, 5-fluoro-deoxyuridine triphosphate; dTMP, deoxythymidine monophosphate; dTTP, deoxythymidine triphosphate; TS, thymidylate synthase.

The European Committee on Antimicrobial Susceptibility Testing (EUCAST) has not set clinical breakpoints for 5-fluorocytosine. To generate MIC data for epidemiologic cutoff (ECOFF) and future breakpoint setting, we included 5-fluorocytosine in our routine EUCAST susceptibility test panels in 2022. The results were somewhat unexpected. All *C. tropicalis* isolates were susceptible to the echinocandins and fluconazole; however, 50% demonstrated high 5-fluorocytosine MICs (>16 mg/L), whereas the remaining *C. tropicalis* isolates had MICs <0.5 mg/L. In comparison, only 0.8% (3/353) of *C. albicans* and 2.2% (5/227) of *C. glabrata* exhibited elevated MICs of 1 to >16 mg/L (Table 2).

**Table 2:** 5-fluorocytosine susceptibility of *Candida* spp. in study of clonal expansion of 5-fluorocytosine–resistant *Candida tropicalis,* Denmark*

**Year sampled **	**MIC, mg/L**												**Total**	**Wild-type UL, mg/L**	**Non–wild-type rate, %**
	<0.016	0.03	0.06	0.125	0.25	0.5	1	2	4	8	16	>16			
*C. tropicalis*															
1998–2004	0	1	2	17	4	0	**0**	**0**	**0**	**0**	**0**	**1**	25	0.5	4.0
2011–12	0	0	12	7	0	0	**0**	**0**	**0**	**0**	**0**	**4**	23	0.5	17.4
2022	1	1	9	18	2	1	**0**	**0**	**0**	**0**	**3**	**29**	64	0.5	50.0
*C. albicans*															
2022	0	2	8	144	154	28	14	**2**	**0**	**0**	**0**	**1**	353	1	0.8
*C. glabrata*															
2022	1	8	86	122	5	**0**	**1**	**1**	**3**	**0**	**0**	**0**	227	0.25	2.2

*The reference strain, *Candida tropicalis* ATCC 750, had a MIC of 0.06 mg/L. Bold text indicates non–wild-type isolates. UL, upper limit.

Few studies have investigated the prevalence of 5-fluorocytosine resistance in *C. tropicalis* across Europe, where resistance has emerged gradually since the 1990s. In 1996, Law et al. reported a prevalence of 17% for 5-fluorocytosine resistance (defined as MIC >8 mg/L) and 37% for of intermediate susceptibility (defined as MIC 2–8 mg/L) in *C. tropicalis* isolates collected over 4 years in northwestern England (*10*). Similarly, Tortorano et al. observed a 30% prevalence of 5-fluorocytosine resistance (MIC >32 mg/L) in bloodstream isolates from 11 different institutions in Lombardy, Italy, during 1997–1999, indicating a widespread, non–hospital-specific prevalence of resistance (*11*). More recently, Desnos-Ollivier et al. reported that 35% of *C. tropicalis* strains from blood cultures collected in France (2002–2006) were resistant to 5-fluorocytosine (MIC >8 μg/mL; as defined by authors) (*12*). Although no mutations were identified in key pyrimidine salvage pathway genes involved in 5-fluorocytosine uptake and metabolism, including *FCY2* (purine–cytosine permease), *FCY1* (cytosine deaminase), and *FUR1* (uracil phosphoribosyl transferase), the authors observed evidence of a clonal spread of resistant strains, particularly in the Paris region. In addition, they identified a consistent correlation between 5-fluorocytosine resistance and a missense mutation (K177E) in the *URA3* (orotidine-5′-phosphate decarboxylase) gene, encoding a key enzyme in the later stages of the de novo pyrimidine biosynthesis. The authors proposed that this mutation could alter the structure and function of the URA3 enzyme, potentially modifying its binding affinity for substrates involved in nucleic acid synthesis. They also suggested that increased expression of *URA3* in these strains could contribute to 5-fluorocytosine resistance, possibly by promoting the overproduction of uridine monophosphate (UMP), a precursor for deoxyribonucleotide synthesis. However, direct evidence linking *URA3* upregulation to the observed resistance phenotype was not provided.

In this study, we aimed to investigate the epidemiology of 5-fluorocytosine resistance in *C. tropicalis* in Denmark over a 20-year perspective. We also explored the underlying molecular mechanisms of 5-fluorocytosine resistance and investigated the genetic similarity between susceptible and 5-fluorocytosine–resistant *C. tropicalis* isolates.

## Materials and Methods

### Isolates

 Our MIC study included all unique clinical *C. tropicalis* (excluding same patient, same species, same 5-fluorocytosine susceptibility isolates within 30 days) received at Statens Serum Institut (Copenhagen, Denmark) in 3 time periods spanning 2 decades: 25 isolates from 1998–2004, 23 isolates from 2011–2012, and 64 isolates from 2022. We selected the duration of each period to include a minimum of 20 isolates per period. Overall, 78% of the isolates derived from blood cultures. We included the *C. tropicalis* reference strain (ATCC 750) as an external comparator. We performed species identification using morphology on CHROMagar Candida (BD BBL; BD, https://www.bd.com) or cornmeal Tween 80 agar (Dalmau technique), or both, with carbohydrate assimilation profiling with ID32C (bioMérieux, https://www.biomerieux.com) for earlier isolates in some cases. In later periods, we used matrix-assisted laser desorption/ionization time-of-flight mass spectrometry (Bruker, https://www.bruker.com), supplemented with internal transcribed spacer sequence analysis when needed.

### Susceptibility Testing and Categorization of Susceptibility

We performed MIC determination in RPMI 1640 medium according to EUCAST E.Def 7.3.2, as previously described (*13*,*14*). We stored 5-fluorocytosine pure substance (Sigma-Aldrich, https://www.sigmaaldrich.com) in aliquots with 5,000 mg/L stock solutions prepared in sterile water at −80°C. The final concentration range was 0.016–16 mg/L. We determined wild-type upper limits (WT-ULs) using the ECOFFinder program with 99.9% of the modeled distributed (https://www.EUCAST.org; accessed 2024 Dec 11) and used to categorize isolates as non–wild-type when MIC >WT-UL (*15*).

### DNA Extraction

We transferred colonies to 400 µL of easyMAG Lysis Buffer (bioMérieux) for DNA extraction using the automated eMAG extraction system (bioMérieux). We eluted genomic DNA in 100 µL of Extraction Buffer 3 (bioMérieux) and stored at −20°C until further processing.

### Molecular Analysis of 5-Fluorocytosine Resistance Mechanisms in *C. tropicalis*

For the molecular studies, we excluded 8 isolates from 2022 for practical reasons (3 susceptible [MIC<0.5 mg/L] and 5 non–wild-type [MIC>0.5 mg/L]). We sequenced the genes *FCY2*, *FCY1*, *FUR1*, and *URA3* for the remaining 104 isolates (72 susceptible and 32 non–wild-type isolates) (Appendix Table 1). We validated redesigned and newly designed primers through in silico analysis for specificity and complementarity using the *C. tropicalis* ATCC 750 genome (https://genomes.atcc.org/genomes) as a reference. PCR cycling conditions were initial denaturation at 95°C for 10 minutes, 35 cycles of denaturation at 95°C for 30 seconds, annealing at the specified temperature for 45 seconds, and extension at 72°C for 90 seconds. We included a final extension at 72°C for 7 minutes before cooling to 8°C. We visualized PCR products on agarose gel, then performed Sanger sequencing (Macrogen Europe, https://www.macrogen-europe.com). We analyzed the sequencing data in the CLC Main Workbench software version 23.0.3 (QIAGEN, https://www.qiagen.com) (Appendix Table 1).

### Genotyping of *C. tropicalis* and Cluster Analysis

We used the microsatellite-based typing method developed by Wu et al. (*16*). We modified primers for amplifying the microsatellite loci and PCR conditions and ran singleplex assays for some primer sets and duplex assays for others (Appendix Table 2). We assembled PCR reactions in a total volume of 25 µL, containing 2.5 µL genomic DNA, 0.4 µM of each duplex primer or 0.8 µM of each singleplex primer, 6 µL of distilled water for the duplex assay or 8 µL for the singleplex assay, and 12.5 µL of Extract-N-amp-PCR ReadyMix (Sigma Aldrich). We performed all PCR reactions using a SimpliAmp Thermal Cycler (ThermoFisher) under the following conditions: initial denaturation at 95°C for 10 minutes, followed by 35 cycles of denaturation at 95°C for 30 seconds, annealing at 52°C for 30 seconds, elongation at 72°C for 90 seconds, and a final extension at 72°C for 7 minutes. We analyzed PCR products on a 2% agarose gel and visualized after staining with ethidium bromide under UV light.

For fragment sizing, we combined 1 µL of each duplex and corresponding singleplex PCR product with 11.2 µL distilled water and 0.8 µL GeneScan 500 ROX, resulting in a total volume of 14 µL. We heated the mixture at 95°C for 3 minutes, cooled on ice, and analyzed using the SeqStudio Genetic Analyzer (Thermo Fisher). We analyzed fluorescent peaks with Peak Scanner software (Thermo Fisher) to determine fragment sizes measured in base pairs. We defined a singleton as a genotype found in a single strain, whereas a cluster refers to a genotype shared by >2 strains. Finally, we illustrated the genetic relationships among isolates by constructing a minimum-spanning tree using BioNumerics software version 8.1.1 (bioMérieux).

## Results

### Susceptibility Epidemiology

Adopting the WT-UL values determined using the 2022 dataset and including 99.9% of the modeled MIC distributions, the 5-fluorocytosine proportion of *C. tropicalis* isolates non–wild-type to 5-fluorocytosine increased exponentially from 4.0% to 50.0% over 2 decades (Table 2). In contrast, the non–wild-type rates in *C. albicans* (0.8%) and *C. glabrata* (2.2%) in 2022 remained low. Focusing on the *C. tropicalis* isolates from 2022, we found no pattern regarding 5-fluorocytosine susceptibility and the geographic origin of the isolates (Appendix Table 3). The comparator control strain (ATCC 750) MIC was 0.06 mg/L.

### Molecular Analysis of Genes Contributing to 5-Fluorocytosine Resistance

We detected no mutations resulting in amino acid alterations in the *FCY1* gene in the non–wild-type or susceptible *C. tropicalis* isolates. However, we identified several point mutations that resulted in amino acid substitutions and nonsense mutations (resulting in truncated proteins), in the *FCY2, FUR1*, and *URA3* genes (Table 3). We found 2 nonsense mutations and 1 nucleotide deletion in *FCY2* exclusively in 5-fluorocytosine non–wild-type isolates; the nonsense mutations caused premature translation termination (E49X and Q7X), and the nucleotide deletion caused a frameshift in the protein sequence (K6NfsX10). Of those mutations, E49X was found in 30 of the 32 non–wild-type *C. tropicalis* isolates. In contrast, the *URA3* mutation leading to a K177E alteration was present in both wild-type and non–wild-type isolates. In addition, missense alterations K5Q and A157S in FUR1, along with various heterozygous mutations in *FCY2,* were found exclusively in wild-type isolates (Table 3). The comparator control strain (ATCC 750) exhibited wild-type alleles for all 4 genes analyzed.

**Table 3:** Overview of target gene mutations found in *Candida tropicalis* isolates in study of clonal expansion of 5-fluorocytosine–resistant *C. tropicalis,* Denmark

Amino acid alteration	Mutation type	No. strains	Total strains, N = 104	Expected effect on protein	Modal MIC, mg/L (range)†
*FCY2*					
E49X	Nonsense (homozygous)	30 (R)	32 (R)	Premature termination, loss of function	>16, non–wild-type
Q7X	Nonsense (homozygous)	1 (R)	32 (R)	Premature termination, loss of function	>16, non–wild-type
K6NfsX10	Single-nucleotide deletion (homozygous)	1 (R)	32 (R)	Frameshift leading to premature termination, loss of function	>16, non–wild-type
E49X & M162I	Heterozygous	12 (S)	72 (S)	No significant impact on transporter function	0.125 (0.06–0.5)
H201I	Heterozygous	1 (S)	72 (S)	No significant impact on transporter function	0.125
I473L	Heterozygous	2 (S)	72 (S)	No significant impact on transporter function	(0.06–0.125)
M130T	Heterozygous	1 (S)	72 (S)	No significant impact on transporter function	0.25
M162I	Heterozygous	1 (S)	72 (S)	No significant impact on transporter function	0.125
S108F	Heterozygous	1 (S)	72 (S)	No significant impact on transporter function	0.06
W67X	Heterozygous	1 (S)	72 (S)	Discrete impact on transporter function	0.5
S258X	Heterozygous	1 (S)	72 (S)	No significant impact on transporter function	0.25
*FUR1*					
K5Q & A157S	Homozygous	1 (S)	72 (S)	No impact on 5-FC conversion	0.125 (0.06–0.5)
*URA3*					
K177E	Homozygous	30 (R) + 2 (S)	32 (R) + 72 (S)	No impact on protein function	0.125 (0.06–0.5)

*Strains were wild-type except as indicated. *FCY2,* purine-cytosine permease; *FUR1*, uracil phosphoribosyltransferase; R, resistant (non–wild-type); S, susceptible (wild-type); *URA3,* orotidine-5'-phosphate decarboxylase †Modal MIC value presented for alterations represented by >10 isolates, MIC range in parentheses for alterations represented by <10 isolates, and MIC value for alterations found in a single isolate. For comparison, modal MIC for wild-type population is 0.125 mg/L.

### Allelic and Cluster Analysis

**Figure 1:**
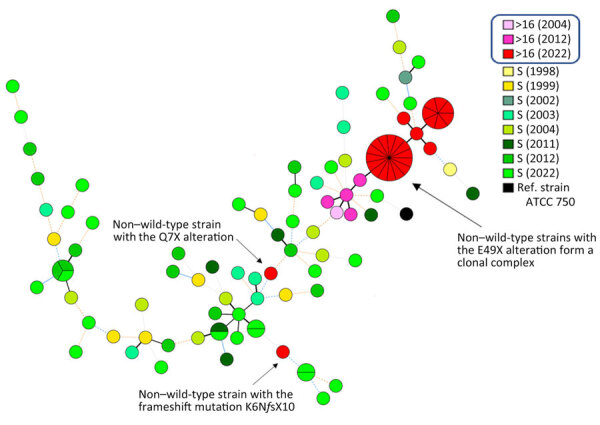
Minimum spanning tree illustrating the genotypic relationships among *Candida tropicalis* isolates, Denmark. Each node represents a distinct genotype; node size is proportional to the number of strains sharing the same allelic profile. Lines connecting nodes indicate genetic differences: thick solid black indicates 1 allele difference; thin solid black, 2 alleles; thin solid blue, 3 alleles; thin dashed blue, 4 alleles; orange dashed, 5 alleles; orange dotted, 6 alleles; and grey dotted, >6 alleles. Node colors represent the year of collection and 5-fluorocytosine resistance status. Non–wild-type strains with the E49X alteration form a clonal complex with minimal genetic variation over a 20-year period. In contrast, isolates carrying the Q7X or K6NfsX10 alterations are genetically unrelated to each other and to other strains, suggesting sporadic acquisition of resistance. Note: Line length does not reflect evolutionary distance. Boxed items at top of key indicate MICs for non–wild-type strains. S, susceptible.

We next explored the genetic relationships among *C. tropicalis* strains in Denmark to determine whether 5-fluorocytosine resistance emerged as a result of clonal spread among hospitalized patients. We identified 66 genotypes among the 72 susceptible strains, comprising 84% of the total observed diversity, including 62 unique singletons and 4 small clusters (Figure 1). In contrast, the 32 genotyped non–wild-type strains accounted for 12 genotypes, forming 2 clusters and 10 distinct singletons. One cluster included 15 non–wild-type strains (47% of all non–wild-type isolates), whereas a smaller cluster contained 7 non–wild-type strains (22%). Eight non–wild-type singletons varied by 1 allele, whereas the remaining 2 were completely distinct (Figure 1). We determined the allelic profiles of the 104 clinical *C. tropicalis* strains, including the profile for the comparator control strain (ATCC 750) (Appendix Table 4).

## Discussion

In this study, we report a concerning exponential 4-fold increase per decade over a 20-year period in 5-fluorocytosine resistance in clinical *C. tropicalis* isolates in Denmark. The underlying molecular mechanism is primarily associated with an E49X alteration in the purine-cytosine permease enzyme (encoded by *FCY2*), which was found exclusively in non–wild-type strains and results in a severely truncated and likely nonfunctional transporter protein. Furthermore, we show that this genotype has expanded clonally in Denmark. Those findings are both surprising and worrying for several reasons. First, 5-fluorocytosine is rarely used in Denmark (<1–4 patients per year according to the national medicine sales registry [https://medstat.dk]). Second, the non–wild-type strains were unique and epidemiologically unrelated, with no geographic pattern in resistance rates across the country, as expected because a previous genotyping study found no evidence of clonal spread of candidemia isolates in Denmark (*17*). This raises the question whether a common source of non–wild-type *C. tropicalis* exists or whether a common selecting factor other than 5-fluorocytosine use in humans has contributed to the observed increase in resistance, either in hospitals or in the environment.

Our sequencing targeted the *FCY2*, *FCY1*, and *FUR1* genes, essential for 5-fluorocytosine uptake and conversion, along with the *URA3* gene involved in UMP biosynthesis. Deleterious mutations were found in *FCY2*-encoding purine-cytosine permease, responsible for the cellular uptake of 5-fluorocytosine and the primary molecular target involved in 5-fluorocytosine resistance. Of note, 92% of non–wild-type strains harbored a G145T nucleotide alteration in both *FCY2* alleles, converting the GAA codon for glutamate (E) to a stop codon (TAA) and resulting in premature termination at position 49 (designated E49X) of the 509-aa sequence. The G145T point mutation has been reported only once, in a study by Chen et al. (2011), where it was observed in 1 resistant isolate among 97 tested clinical strains (*18*). Our data strongly suggest that the G145T point mutation is a key mechanism behind 5-fluorocytosine resistance in *C. tropicalis* isolates in Demark.

Two non–wild-type strains did not contain the E49X alteration. One of those displayed a novel nonsense mutation, termed Q7X, caused by the C19T nucleotide substitution in both *FCY2* alleles. The other non–wild-type strain harbored a novel frameshift mutation caused by the deletion of an adenine (A) at position 18 of the *FCY2* sequence, designated K6NfsX10. This frameshift mutation substitutes a lysine (K) at position 6 to asparagine (N) and results in premature termination of translation after 10 aa. Each of those 3 mutations leads to the early truncation of the FCY2 protein, likely rendering it deficient in critical functional domains essential for proper transporter activity, including substrate binding, cofactor interactions, and cellular localization.

We did not detect amino acid substitutions in cytosine deaminase (FCY1), in either non–wild-type or susceptible *C. tropicalis* isolates. However, 1 susceptible isolate harbored 2 missense mutations in *FUR1*, leading to K5Q and A157S alterations in uracil phosphoribosyl transferase. The absence of mutations in genes (such as *FCY1* and *FUR1*) downstream of 5-fluorouracil, the active metabolite of 5-fluorocytosine, in non–wild-type isolates suggests that 5-fluorouracil, a chemotherapeutic agent (*8*), does not play a significant role in conferring resistance to 5-fluorocytosine in *C. tropicalis*. Furthermore, 5-fluorouracil does not use the same transporter protein (FCY2) as 5-fluorocytosine (*19*). Taken together, those findings indicate that the selection pressure driving 5-fluorocytosine resistance in *C. tropicalis* likely occurred upstream of 5-fluorouracil metabolism. However, other purine analogs that also rely on FCY2 for cellular entry may contribute to resistance emergence. Such agents, whether used as chemotherapies or in other medical or environmental contexts, could impose selective pressure on fungal populations, potentially promoting mutations in the transporter protein that confer 5-fluorocytosine resistance. Those results further suggest the existence of an alternative cytosine salvage pathway in *C. tropicalis*, highlighting its capacity for survival and proliferation in environments with fluctuating drug exposure, both in clinical and environmental contexts.

**Figure 2:**
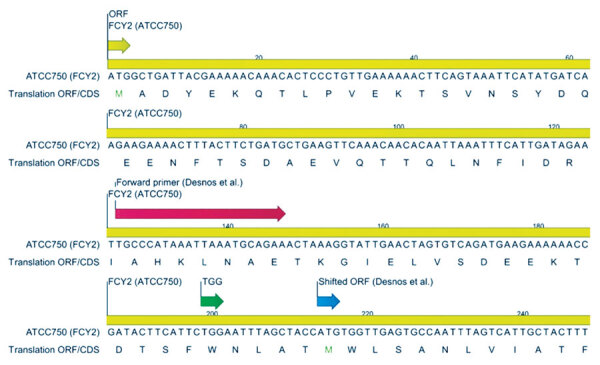
*FCY2* sequence encoding the purine-cytosine permease derived from contig 14 of the *Candida tropicalis* strain ATCC 750 genome assembly, published on the ATCC Genome Portal in 2020. The coding sequence spans positions 2152980–2154500. The ORF is depicted at the top left of the figure; long red arrow illustrates the *FCY2* forward primer designed by Desnos-Ollivier et al. (*12*), and the blue arrow indicates the ORF they used. The green arrow represents the tryptophan codon TGG, which is converted to stop codon TGA in the MYA 3404 genome assembly. CDS, coding sequence; ORF, open reading frame.

Desnos-Olivier et al. (*12*) reported a 35% 5-fluorocytosine resistance rate in *C. tropicalis* in the Paris area in 2002–2006, dominated by a clonal population carrying a K177E alteration in orotidine-5′-phosphate decarboxylase (encoded by *URA3*) but no mutations in the *FCY2* gene when compared with the wild-type strain. The authors proposed that increased *URA3* expression, driven by the K177E mutation, could lead to overproduction of UMP by upregulating the pyrimidine biosynthesis pathway, contributing to 5-fluorocytosine resistance. However, considering the high catalytic efficiency of orotidine-5′-phosphate decarboxylase across many organisms and the fact that pyrimidine nucleotide biosynthesis is primarily regulated at earlier steps in the pathway, it seems unlikely that the K177E mutation alone would substantially alter pyrimidine nucleotide pools and drive resistance (*20*–*22*). Although we detected the K177E alteration in all 5-fluorocytosine–non–wild-type isolates from Denmark harboring the nonsense mutation (E49X), the mutation was also present in 2 susceptible isolates, bringing into question its proposed role in resistance. We performed in silico analysis of the *FCY2* forward primer used in the France study and confirmed complementarity to the target gene. However, we noted that the forward primer annealed 126 bp downstream of the open reading frame (Figure 2). This primer positioning likely led to incomplete sequencing of the *FCY2* coding region, missing potential point mutations in the early region of the gene. We speculate that this positioning may be a result of using the *FCY2* reference sequence derived from the genome assembly of *C. tropicalis* MYA 3404 (BioProject no. PRJNA13675). Our in silico analysis indicates a possible mutation or sequencing error in this assembly, leading to a premature TGA stop codon instead of the TGG tryptophan codon, 199 bp downstream of the open reading frame (Figure 2). Moreover, analysis of the more recent *C. tropicalis* ATCC750 genome assembly (2020) confirmed the presence of the TGG codon with no premature stop codon observed at this position. Finally, our sequencing data of both non–wild-type and susceptible 5-fluorocytosine strains was consistent with the updated reference sequence, supporting our observation. Resequencing of the *FCY2* gene of the strains from France using updated primers and reference sequence would be of interest.

Our sequencing analysis revealed a significant level of heterozygosity across all strains; 12/72 (16.7%) of the 5-fluorocytosine–susceptible population carrying the G145T point mutation (causing E49X in homozygous isolates) in 1 of the 2 *FCY2* alleles. This finding may indicate an increased likelihood of resistance developing through a single genetic rearrangement (*18*,*23*).

In contrast to the broad genetic diversity observed among susceptible strains, the genotyping dataset highlighted the close genetic relatedness among the non–wild-type strains harboring the E49X alteration, with only 1 allele difference between the early 2004 non–wild-type isolate and subsequent strains collected over the next 20 years. This finding supports the hypothesis that this specific non–wild-type genotype, likely introduced or emerged in Denmark 20 years ago, has undergone gradual microevolution and effectively spread among hospitalized patients or in the population in general. In contrast, the 2 remaining non–wild-type isolates (1 carrying Q7X alterations and the other K6Nf*s*X10) were not related to each other or to the remaining non–wild-type and susceptible isolates, suggesting sporadic resistance acquisition. Given that 5-fluorocytosine is rarely used in Denmark and extremely rarely for *C. tropicalis* infections, the drivers of the clonal expansion of the 5-fluorocytosine clone remain unexplained. Those findings call for studies on potential unrecognized selection factors in human medicine, environmental influences, and fungus-specific factors such as virulence, biofilm formation, or surface adherence.

In conclusion, our study provides new knowledge on the molecular mechanisms underlying 5-fluorocytosine resistance in *C. tropicalis* strains and documents a clonal spread along with notable increase in resistance prevalence among *C. tropicalis* isolates in the absence of a recognized selection pressure in Denmark. Our findings present a challenge in healthcare facilities, requiring attention, surveillance, improved infection control measures, and collaborative efforts to clarify drivers of resistance and develop effective prevention strategies.

Appendix Additional information about exponential clonal expansion of 5-fluorocytosine–resistant *Candida tropicalis* and underlying molecular mechanisms.
