# Supplementary material for: Exponential Clonal Expansion of 5-Fluorocytosine–Resistant Candida tropicalis and New Insights into Underlying Molecular Mechanisms
Source: Emerg Infect Dis. 2025 May;31(5):977–85. doi: 10.3201/eid3105.241910 (PMC12044241; doi:10.3201/eid3105.241910)
Supplement: Appendix — Additional information about exponential clonal expansion of 5-fluorocytosine–resistant Candida tropicalis and underlying molecular mechanisms. [file 24-1910-Techapp-s1.pdf]

Article DOI: https://doi.org/10.3201/eid3105.241910

EID cannot ensure accessibility for supplementary materials supplied by authors. Readers who have difficulty accessing supplementary content should contact the authors for assistance.

Exponential Clonal Expansion of 5-Fluorocytosine–Resistant *Candida tropicalis* and New Insights into Underlying Molecular Mechanisms

Appendix

Appendix Table 1. Amplification and sequencing primers of genes associated with 5-FC resistance*

Primer	Sequence (5'→3')	Purpose	Tm°C	Ref. sequence	Source
URA3-F (+)	ATTGAAGAAAGAGTTTATGGAA	PCR	51°C	AF040702†	Own design
URA3-S1 (+)	ATTGGATAGTCCCTCTAAACTCACTACTA	Seq			Desnos-Ollivieret al.
URA3-S2 (-)	AATCAACTATTCAAGTTGACCG	Seq			Desnos-Ollivieret al.
URA3-S3 (+)	TGCCGATATTGGAAATACAGTTA	Seq			Desnos-Ollivieret al.
URA3-S4 (-)	AGCATTAGTTATATCACTCCATGATGAA	Seq			Desnos-Ollivieret al. (modified)
URA3-R (-)	TAACAGTAATAATCAACTATTCAAGT	PCR			Own design
FCY1-F (+)	GGTTACTCTGAAGGAGGTATT	PCR&seq	51°C	CTRG_02927†	Own design
FCY1-R (-)	CCTACAAATACATTATCCTTCA	PCR&seq			Own design
FCY2-F1 (+)	CCATCGCAAATAAAAAGTCAA	PCR&seq	56°C	HQ166001†	Own design
FCY2-R1 (-)	GGAAGCAACAAACCCAAAA	Seq			Desnos-Ollivieret al.
FCY2-F2 (+)	TGCTGCCGATTATGTTGTTT	Seq			Desnos-Ollivieret al.
FCY2-R2 (-)	GTGAAAACGAGCCAATCCA	PCR&seq			Desnos-Ollivieret al. (modified)
FUR1-F (+)	TAGTAATCAAATCCTCTCGCC	PCR&seq	56°C	CTRG_02689†	Own design
FUR1-R (-)	GTGTATGTAGTGATAATTGCTATGC	PCR&seq			Desnos-Ollivieret al. (modified)

* FCY1, cytosine deaminase (pyrimidine salvage pathway); FCY2, purine-cytosine permease (carrier protein); FUR1, Uracil phosphoribosyltransferase (pyrimidine salvage pathway); URA3, orotidine-5'-phosphate decarboxylase (de novo pyrimidine biosynthesis pathway). (+), the 5´primer or the forward primer annealing to the sense strand of the gene; (-), the 3´primer or the reverse primer annealing to the anti-sense strand of the gene.
†The sequence was verified by alignment with the genome assembly of *C. tropicalis* strain ATCC 750 and found to be error-free.

Appendix Table 2. Primers' specifications of microsatellite analysis (MLVA) of *Candida tropicalis.* The primers were adapted with slight modifications from the method developed by Wu et al. (2014), serving as a reference for this study.

MLVA primer	Sequence (5'→3')	Repetitive motif of STRs	Tm °C	Type of assay
Di-nucleotide markers				
Ctrm12-F	**FAM**-TGTGTGTCTATTACCTCCCA	(AC)	51	Duplex
Ctrm12-R	CTGTCAGTTGTACATCATCG		48	Duplex
Ctrm21-F	**HEX**-GTGTCTTGTAAAAGCCACC (mod.)	(TG)	50	Duplex
Ctrm21-R	GGATTACTGGACTTGACCTG		52	Duplex
Ctrm24-F	**NED**-ACAACTACTGACATCCCAGC	(TA)	52	Singleplex
Ctrm24-R	CTTCAGTATTCACCCCTTTC		51	Singleplex
Tri-nucleotide markers				
Ctrm1-F	**FAM**-GCAACAGTTGATAGATCAAGC (mod.)	(AGA)	53	Duplex
Ctrm1-R	ACGAACTATCACTTTTAGGAGT (mod.)		50	Duplex
Ctrm10-F	**HEX**-AGTTTTCCTGTTGCTGGT (mod.)	(ATG)	49	Duplex
Ctrm10-R	CATTGAGATTGGAAGAAGTG (mod.)		50	Duplex
Ctrm28-F	**NED**-TAGTTCGAATTTGTTTGGAT	(TTA)	50	Singleplex
Ctrm28-R	GTAAAGTCACGGGGTATTGT		52	Singleplex

Appendix Table 3. Geographic distribution of *Candida tropicalis* strains collected in 2022, categorized by susceptibility (S) and resistance (R) to 5-fluorocytosine

Geographic origin	Susceptible (S)	Resistant (R)	Total
Copenhagen RH	16	13	29
Greater Copenhagen Hv.	1	5	6
Greater Copenhagen He.	7	4	11
Zeeland Sl.	2	2	4
Funen OUH	2	3	5
Jutland Ve.	1	0	1
Jutland AUH	3	5	8
Total	32	32	64

Publisher: CDC; Journal: Emerging Infectious Diseases
Article Type: Research; Volume: 31; Issue: 5; Year: 2025; Article ID: 24-1910
DOI: 10.3201/eid3105.241910; TOC Head: Research

Appendix Table 4. Allelic profiles and susceptibility patterns of clinical *Candida tropicalis* strains*

Genotype no.	No. isolates	5-FC	Type of genotype	Ctrm12		Ctrm21		Ctrm24		Ctrm1		Ctrm10		Ctrm28	
				I	II	I	II	I	II	I	II	I	II	I	II
1	1	S	Singleton	237	237	331	331	450	450	390	390	321	315	397	397
2	1	S	Singleton	237	239	329	329	450	450	390	390	321	321	397	397
3	1	S	Singleton	237	237	328	354	450	450	373	390	321	321	416	416
4	3	S	Cluster	245	260	328	354	450	450	385	385	321	334	397	397
5	1	S	Singleton	245	260	328	354	450	450	385	390	321	334	397	397
6	1	S	Singleton	245	260	329	354	450	450	385	385	321	321	397	397
7	1	S	Singleton	260	260	328	328	450	450	385	385	321	321	397	397
8	1	S	Singleton	245	260	328	328	450	450	385	385	321	347	403	403
9	1	S	Singleton	239	239	329	331	450	458	400	400	321	356	397	397
10	1	S	Singleton	249	255	354	354	452	452	409	448	321	343	397	397
11	1	S	Singleton	243	243	335	337	450	450	396	433	321	337	400	400
12	1	S	Singleton	239	243	329	331	450	450	393	406	321	465	400	400
13	1	S	Singleton	243	253	330	354	450	450	376	472	321	340	400	400
**14**	**15**	**R**	**Cluster**	**235**	**235**	**341**	**341**	**450**	**450**	**373**	**373**	**321**	**318**	**400**	**400**
**15**	**1**	**R**	**Singleton**	**235**	**235**	**341**	**341**	**450**	**450**	**373**	**373**	**321**	**324**	**400**	**400**
**16**	**1**	**R**	**Singleton**	**235**	**235**	**341**	**341**	**450**	**450**	**373**	**373**	**321**	**321**	**400**	**400**
**17**	**1**	**R**	**Singleton**	**235**	**243**	**341**	**341**	**450**	**450**	**373**	**373**	**321**	**318**	**400**	**400**
**18**	**7**	**R**	**Cluster**	**235**	**235**	**341**	**343**	**450**	**450**	**373**	**373**	**321**	**324**	**400**	**400**
**19**	**1**	**R**	**Singleton**	**235**	**235**	**330**	**341**	**450**	**450**	**373**	**373**	**321**	**324**	**400**	**400**
**20**	**1**	**R**	**Singleton**	**235**	**243**	**330**	**341**	**450**	**450**	**373**	**376**	**321**	**318**	**400**	**400**
**21**	**1**	**R**	**Singleton**	**235**	**243**	**330**	**341**	**450**	**450**	**373**	**373**	**321**	**318**	**400**	**400**
**22**	**1**	**R**	**Singleton**	**235**	**243**	**330**	**341**	**450**	**450**	**373**	**373**	**321**	**321**	**400**	**400**
**23**	**1**	**R**	**Singleton**	**235**	**258**	**330**	**341**	**450**	**450**	**373**	**373**	**321**	**318**	**400**	**400**
24	1	S	Singleton	243	262	331	331	450	450	373	373	321	321	400	400
25	1	S	Singleton	255	255	328	328	450	450	373	373	321	315	400	400
26	1	S	Singleton	258	258	354	354	448	450	373	373	340	347	400	400
27	1	S	Singleton	258	258	356	356	450	450	373	373	340	340	400	400
28	1	S	Singleton	253	256	354	354	450	450	373	373	340	347	394	394
29	1	S	Singleton	260	260	354	354	450	450	373	373	340	347	394	394
30	1	S	Singleton	253	260	354	354	450	450	373	373	340	340	400	400
31	1	S	Singleton	260	260	354	354	450	450	373	373	340	340	400	400
32	1	S	Singleton	245	245	354	354	450	450	373	373	303	303	382	382
33	1	S	Singleton	256	256	330	330	448	450	373	373	303	324	379	400
34	1	S	Singleton	258	258	330	330	448	450	373	373	303	324	379	400
35	1	S	Singleton	256	256	330	337	450	450	390	393	315	324	400	400
36	1	S	Singleton	233	253	326	350	450	450	373	373	309	309	397	397
37	3	S	Cluster	251	251	354	354	448	448	373	373	321	321	403	403
38	1	S	Singleton	251	255	354	354	448	448	373	373	321	321	403	403
39	1	S	Singleton	255	269	354	354	448	448	373	373	321	321	403	403
40	1	S	Singleton	251	253	352	354	448	448	373	373	321	321	403	403
41	2	S	Cluster	253	253	354	354	448	448	373	373	321	321	403	403
42	1	S	Singleton	253	253	354	354	448	450	373	373	321	321	403	403
43	1	S	Singleton	251	250	350	354	448	450	373	373	321	340	403	403
44	1	S	Singleton	251	250	354	354	448	450	373	373	321	340	403	403
45	1	S	Singleton	253	253	350	354	448	448	376	376	321	321	403	403

Genotype no.	No. isolates	5-FC	Type of genotype	Ctrm12		Ctrm21		Ctrm24		Ctrm1		Ctrm10		Ctrm28	
				I	II	I	II	I	II	I	II	I	II	I	II
**46**	**1**	**R**	**Singleton**	**247**	**260**	**339**	**354**	**448**	**450**	**373**	**373**	**340**	**340**	**403**	**403**
47	1	S	Singleton	241	249	328	330	448	452	373	373	327	340	403	403
48	1	S	Singleton	233	233	356	356	450	450	373	373	303	303	403	403
49	1	S	Singleton	233	233	356	356	450	450	373	373	303	321	403	403
50	1	S	Singleton	235	235	356	356	450	450	373	373	321	303	403	403
51	1	S	Singleton	233	253	329	329	450	450	373	373	340	353	403	403
52	1	S	Singleton	243	243	354	368	450	450	373	373	321	340	403	403
53	1	S	Singleton	241	241	328	328	450	450	442	442	321	318	403	403
54	1	S	Singleton	239	241	328	354	450	450	367	367	321	315	403	403
55	1	S	Singleton	237	237	328	328	450	450	445	445	321	324	403	403
56	1	S	Singleton	243	255	328	331	450	454	373	382	321	318	403	403
57	1	S	Singleton	237	253	331	331	450	450	393	393	321	321	403	403
58	1	S	Singleton	237	253	331	331	450	450	393	498	321	321	403	403
59	1	S	Singleton	253	253	331	331	450	450	393	466	321	321	403	403
60	1	S	Singleton	267	267	330	330	458	458	373	373	321	303	382	382
61	1	S	Singleton	237	239	331	354	450	450	390	415	315	356	403	422
62	1	S	Singleton	239	239	331	331	444	450	373	390	331	356	403	403
63	1	S	Singleton	239	260	331	354	444	450	412	415	334	340	400	400
64	1	S	Singleton	239	239	331	331	448	457	373	456	456	456	400	400
65	1	S	Singleton	235	255	330	348	448	452	379	393	321	321	394	403
66	1	S	Singleton	235	255	330	348	452	452	382	393	321	321	403	403
67	1	S	Singleton	239	245	330	330	448	452	388	393	321	318	412	412
68	2	S	Cluster	237	249	356	358	448	448	379	393	321	334	406	406
**69**	**1**	**R**	**Singleton**	**237**	**251**	**354**	**356**	**448**	**448**	**379**	**379**	**321**	**334**	**406**	**406**
70	1	S	Singleton	237	250	356	358	448	450	388	393	321	321	406	406
71	1	S	Singleton	251	250	358	358	448	450	379	379	321	334	406	406
72	1	S	Singleton	239	249	330	330	452	456	373	456	337	368	397	397
73	1	S	Singleton	239	249	330	339	450	450	373	400	337	356	391	391
74	1	S	Singleton	229	245	330	360	444	450	396	396	337	337	397	397
75	1	S	Singleton	237	245	328	330	450	450	436	436	331	331	450	454
76	1	S	Singleton	237	245	328	330	450	454	400	439	315	331	406	406
77	1	S	Singleton	237	250	328	328	454	454	400	439	324	324	397	403
78	1	S	Singleton	237	253	328	328	450	452	439	439	315	324	397	403
(ATCC750)	1	S	Singleton	245	245	328	328	456	456	376	376	400	400	400	400

*This table presents allelic profiles of 104 clinical *Candida tropicalis* strains analyzed in the present study, including the profile obtained for the positive control (ATCC750). The profiles were determined by the analysis of six short tandem repeat markers using MLVA. It specifies the frequency of each profile, genotype types, and their respective susceptibility patterns of 5FC. S: susceptible; R: resistant (text in bold); Singleton refers to a genotype represented by a single strain within the studied population; Cluster refers to a genotype shared by two or more strains.
